# Calculation of therapeutic activity of radioiodine in Graves’ disease by means of Marinelli’s formula, using technetium (^99m^Tc) scintigraphy

**DOI:** 10.1007/s12020-016-1074-7

**Published:** 2016-08-24

**Authors:** Piotr Szumowski, Małgorzata Mojsak, Saeid Abdelrazek, Monika Sykała, Anna Amelian-Fiłonowicz, Dorota Jurgilewicz, Janusz Myśliwiec

**Affiliations:** Department of Nuclear Medicine, Medical University of Bialystok, M. Skłodowskiej-Curie St. 24A, 15–276 Bialystok, Poland

**Keywords:** Marinelli’s formula, Thyroid iodine uptake, Thyroid technetium uptake, Graves’ disease

## Abstract

The therapeutic activity of ^131^I administered to patients with Graves’ disease can be calculated by means of Marinelli’s formula. The thyroidal iodine uptake (^131^IU_max_) needed for the calculation is usually determined with the use of ^131^I. The purpose of the paper was to estimate ^131^IU_max_ on the basis of technetium uptake in the thyroid at 20 min (^99m^TcU_20min_). Eighty patients suffering from Graves’ disease were qualified for radioiodine therapy with measurement of fT_4_, fT_3_, thyroid-stimulating hormone and its receptor (TRAb). Prior to the treatment, all the patients were euthyroid. ^131^IU_max_ for each patient was determined according to the levels of ^131^I after 24 h (^131^IU_24h_), while effective half-life (*T*_eff_) according to the measurements of ^131^IU_24h_ and ^131^I uptake after 48 h (^131^IU_48h_). Additionally, on the day before measuring ^131^IU_24h_, ^99m^TcU_20min_ was calculated for each patient. It was demonstrated that there existed a correlation, with statistical significance at *p* < 0.05, between the following pairs of values: TRAb and ^131^IU_24h_, TRAb and ^99m^TcU_20min_, and ^99m^TcU_20min_ and ^131^IU_24h_. The interdependence between ^131^IU_24h_ and ^99m^TcU_20min_ at the level of significance *p* < 0.05 is described by the following algorithms: ^131^IU_24h_ = 17.72 × ln (^99m^TcU_20min_) + 30.485, if TRAb < 10 IU/ml, and ^131^IU_24h_ = 18.03 × ln (^99m^TcU_20min_) + 38.726, if TRAb > 10 IU/ml. It is possible to predict thyroid iodine uptake ^131^IU_24h_ in Graves’ disease on the basis of measuring the uptake of ^99m^TcU_20min_. This shortens the time necessary for diagnosis and enables the calculation of ^131^I activity using Marinelli’s formula.

## Introduction

Radiotherapy is, apart from pharmacotherapy and surgical intervention, one of the major methods of treatment for Graves’ disease (GD). Effective radioiodine therapy for GD consists in calculating an appropriate activity of the radioisotope in order to achieve the highest possible therapeutic efficacy, with the lowest possible radiation of the thyroid, and thus of the patient. The clinics that offer radioiodine therapy in Europe and around the world use various methods of selecting correct therapeutic activities of ^131^I. Our department applies the Marinelli formula, which is recommended by the European Association of Nuclear Medicine (EANM) [1]. Radioiodine ^131^I is usually used to determine the thyroidal iodine uptake required by the formula. Some also use the iodine isotope ^123^I, although much less frequently because of the high costs of its production in a cyclotron [2, 3]. The disadvantages of using ^131^I for therapeutic purposes include: beta radiation during radioactive decay, relatively high gamma radiation energy (364 keV) and the long time (usually 24 h) that is required for assessing the maximum iodine uptake in the thyroid. Technetium ^99m^Tc is another isotope which, like ^131^I, is absorbed by thyrocytes, thanks to sodium/iodide symporter (NIS) proteins. Contrary to ^131^I, it is a pure emitter of gamma radiation, with a much shorter half-life (*T*_1/2_) of 6.04 h (8 days in the case of ^131^I). Moreover, the gamma radiation of technetium carries far less energy than that of iodine (140 keV). Also the time needed for the assessment of its maximum uptake by the thyroid is much shorter than in the case of ^131^I and equals only 20 min. This allows to diminish the time of thyroid diagnostic. As far as their metabolism in thyroid cells is concerned, a major difference between ^99m^Tc and ^131^I is that the former does not participate in the synthesis of hormones. Nevertheless, the thyroidal uptake of both ^9m^Tc and ^131^I in GD depends primarily on the level of thyroid-stimulating hormone (TSH) receptor antibodies (TRAbs), which stimulate the NIS protein to active transport of isotopes into thyrocytes, as well as by intercellular metabolism [4, 5]. The question arises, therefore, whether the assessment of thyroid iodine uptake needed for Marinelli’s formula could be performed with the help of ^99m^Tc. Thus the aims of this study were as follows:

1. An attempt to create an algorithm to assess thyroidal iodine uptake in GD patients qualified for radioactive iodine therapy on the basis of ^99m^Tc scintigraphy of the thyroid.
2. Transformation of Marinelli’s formula used for calculating therapeutic activities of ^131^I by substituting thyroid iodine uptake with thyroid ^99m^Tc uptake.

## Materials and methods

The study sample comprised 80 GD patients: 61 women and 19 men. All the subjects had been referred to the Department of Nuclear Medicine with a view to treating hyperthyroidism, following unsuccessful attempts at pharmacotherapy with thyrostatic drugs (the treatment periods being no shorter than 1.5 years). Prior to the administration of a therapeutic dose of ^131^I, each patient had undergone routine eligibility screening, involving an initial measurement of the thyrotropic hormone, TSH (reference range 0.3–4.0 µIU/ml), free thyroxine, fT_4_ (reference range 0.71–1.85 ng/dl) and free triiodothyronine, fT_3_ (reference range 1.45–3.48 pg/mL), using the immunoenzymatic method, Microparticle Enzyme Immunoassay (Abbott Park, USA), and TSH-TRAb (normal < 2 IU/l) using the radioimmunological method (TRAK Human BRAHMS, Germany). Subsequently it was decided that further diagnostics could only involve those subjects who were euthyroid. Hyperthyroid patients continued to be treated with thyrostatics until euthyroid, up to 2 days before the study.

Next, the 24- and 48-h thyroidal iodine uptake tests were carried out after per os (fasting) administration of a capsule containing ^131^I with the activity of 4 MBq. Additionally, 1 day before the IU_24h_, scintigrams of the thyroid glands were performed, following an intravenous administration of 80 MBq ^99m^Tc. The readouts of thyroid-absorbed radiation began at 20 min of the administration of the radio marker. Both the iodine uptake tests and the technetium scintigraphy estimating the marker’s uptake in the gland were performed with a gamma-camera (NuclineTM Th, Mediso, Hungary), in accordance with the standard procedures. The volume of the thyroid (*m*) was estimated by means of a ultrasonography (USG). USG device was equipped with a 12 L linear transducer (LOGIQ S8, GE Healthcare, USA).

The required therapeutic activity of ^131^I (*A*) was calculated with Marinelli’s formula [6]:

$$A = \frac{{25 \cdot m \cdot D}}{{I{U_{24h}} \cdot {T_{eff}}}}$$

where

A—^*131*^ *I therapeutic activity (MBq)*

25—*unit conversion coefficient*

*m*—*mass of thyroid gland calculated with USG (g)*

D—*absorbed dose of* ^*131*^ *I (Gy), with D* = *150 Gy, as recommended by EANM for GD* [1]

*IU*_*24h*_—*24-h* ^*131*^ *I uptake (%)*

*T*_eff_—*effective* ^*131*^ *I half-life in thyroid gland (days) calculated with gamma-camera based on* ^*131*^ *IU*_*24h*_ *and* ^*131*^ *IU*_*48h*_

The study was approved by the Ethics Committee for Medical Research, Medical University, and is compliant with the good clinical practice guidelines. Informed consent was given by all patients participating in the study.

### Statistical analysis

The statistical analysis of the results of the study was conducted using the software package Statistica 10 (Stat Soft, Tulsa, USA).

A multivariate analysis of variance was used to investigate the influence of fT_4_, fT_3_, TSH, *m* and TRAb on IU_24h_, *T*_eff_ and ^99m^TcU_20min_. The significance level was *p* < 0.05.

The non-linear regression function was used to establish the correlation of the IU_24h_, ^99m^TcU_20min_ and TRAb parameters, with the level of significance at *p* < 0.05.

## Results

It can be noticed among the parameters measured in GD patients in the course of the eligibility study, the effective ^131^I half-life in the thyroid gland (*T*_eff_) has a very low standard deviation (±0.04) and that its mean value equals 5.5 days, while the serum level of thyroid hormones in all the patients before treatment remains within the normal limits, thanks to an effective therapy with thyrostatic medications (Table 1).

**Table 1:** Parameters measured during eligibility screening of GD patients to be treated with ^131^I

	TSH (µIU/ml)	fT_3_ (pg/ml)	fT_4_ (ng/dl)	TRAb (IU/l)	*m* (g)	^99m^TcU_20min_ (%)	IU_24h_ (%)	*T*_eff_ (days)
Mean	1.34	2.54	1.21	10.9	31.6	2.3	45	5.5
Standard deviation	±0.19	±0.36	±0.4	±14.8	±18.6	±3.4	±15.8	±0.04
Maximum	4.67	3.31	1.41	39.7	64.3	8.1	68	5.9
Minimum	0.54	1.54	1.01	1.7	19.4	1.1	32	5.17

*fT*_*4*_ free thyroxine, *fT*_*3*_ free triiodothyronine, *m* mass of thyroid, ^*99m*^ *TcU*_*20min*_ 20-min ^99m^Tc uptake, *IU*_*24h*_ 24-h ^131^I uptake, *T*_eff_ effective ^131^I half-life in thyroid gland, *TRAb* antibodies against TSH receptor, *TSH* thyroid-stimulating hormone

Comparison of the parameters measured in the patients during the eligibility screening period demonstrates that there exists a statistically significant (*p* < 0.05) correlation between the following pairs: TRAb and ^131^IU_24h_, TRAb and ^99m^TcU_20min_, ^99m^TcU_20min_ and ^131^IU_24h_ (Table 2).

**Table 2:** Coefficients of correlation between parameters

	TSH (µIU/ml)	fT_4_ (ng/dl)	fT_3_ (pg/ml)	*m* (g)	TRAb (IU/l)	^99m^TcU_20min_ (%)	^131^IU_24h_ (%)	*T*_eff_ (days)
TSH (µIU/ml)								
fT_4_ (ng/dl)	*r* = 0.56							
	(*p* = 0.072)							
fT_3_ (pg/ml)	*r* = 0.43	*r* = 0.59						
	(*p* = 0.091)	(*p* = 0.301)						
*m* (g)	*r* = 0.24	*r* = 0.31	*r* = 0.28					
	(*p* = 0.086)	(*p* = 0.201)	(*p* = 0.501)					
TRAb (IU/l)	*r* = 0.2	*r* = 0.43	*r* = 0.35	*r* = 0.23				
	(*p* = 0.123)	(*p* = 0.078)	(*p* = 0.101)	(*p* = 0.134)				
^99m^TcU_20min_ (%)	*r* = 0.34	*r* = 0.56	*r* = 0.66	*r* = 0.37	*r* = 0.70^a^			
	(*p* = 0.061)	(*p* = 0.058)	(*p* = 0.069)	(*p* = 0.068)	(*p* = 0.013)			
^131^IU_24h_ (%)	*r* = 0.57	*r* = 0.54	*r* = 0.51	*r* = 0.32	*r* = 0.73^b^	*r* = 0.89^c^		
	(*p* = 0.056)	(*p* = 0.059)	(*p* = 0.059)	(*p* = 0.081)	(*p* = 0.021)	(*p* = 0.031)		
*T*_eff_ (days)	*r* = 0.47	*r* = 0.61	*r* = 0.46	*r* = 0.198	*r* = 0.28	*r* = 0.31	*r* = 0.5	
	(*p* = 0.057)	(*p* = 0.061)	(*p* = 0.072)	(*p* = 0.203)	(*p* = 0.078)	(*p* = 0.099)	(*p* = 0.105)	

Abbreviations: see Table 1

^a,b,c^ Correlation coefficients occurring with statistical significance of *p* < 0.05

The other correlation coefficients in the table do not meet the *p* < 0.05 condition

The statistical analysis revealed that the highest correlation between ^99m^Tc and ^131^I occurs when the TRAb titre is either <10 IU/ml or >10 IU/ml.

**Fig. 1:**
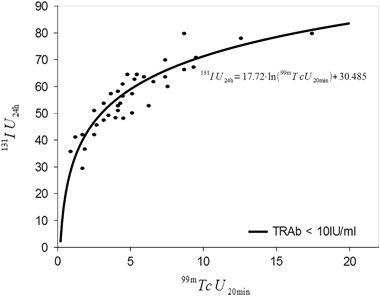
Scatter graph for variables ^99m^TcU_20min_ and ^131^IU_24h_, with TRAb < 10 IU/ml, and regression equation illustrating their relationship

**Fig. 2:**
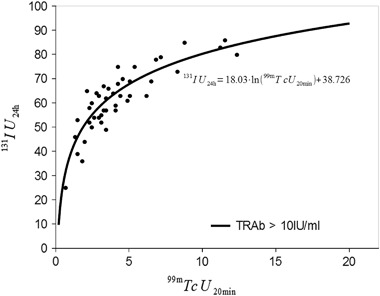
Scatter graph for variables ^99m^TcU_20min_ and ^131^IU_24h_, with TRAb > 10 IU/ml, and regression equation illustrating their relationship

Using this information, further analysis involved the non-linear regression model in order to generate a mathematical formula that would describe the relationship between ^131^IU_24h_ and ^99m^TcU_20min_ for the two ranges of TRAb titres, assuming the significance at *p* < 0.05. It stems from the calculations (Figs. 1 and 2) that the regression model that predicts the dependence of IU_24h_ on ^99m^TcU_20min_, if TRAb < 10 IU/ml, looks as follows:

$$^{131}{\rm IU}_{{\rm 24h}} = 17.72 \times {\rm ln}({99\rm m} {\rm TcU}_{\rm 20min}) + 30.485$$

whereas if TRAb > 10 IU/ml:

$$^{131}{\rm IU}_{\rm 24h} = 18.03 \times {\rm ln}({\rm 99m}{\rm TcU}_{\rm 20min}) + 38.726$$

By substituting ^131^IU_24h_ in the Marinelli formula with the above correlations, and *T*_eff_ with the number 5.5 (the value of *T*_eff_ in all the patients equalled approximately 5.5; see Table 1), we will obtain transformed formulas that can be used to calculate the therapeutic activity of ^131^I on the basis of thyroid ^99m^TcU_20min_ uptake.

If TRAb < 10 IU/ml, the transformed Marinelli’s formula takes this form:

$$A\left( {\rm{MBq}} \right) = \frac{{25 \times m({\rm{g}}) \times D({\rm{G}})}}{{17.72 \times [{\rm{ln}}({}{{\rm{99m}}}{\rm{Tc}}{{\rm{U}}_{{\rm{20min}}}}) + 30.485] \times 5.5}}$$

whereas if TRAb > 10 IU/ml, it looks as follows:

$$A({\rm{MBq}}) = \frac{{25 \times m({\rm{g}}) \times D({\rm{G}})}}{{18.03 \times [{\rm{ln}}({}{{\rm{99m}}}{\rm{Tc}}{{\rm{U}}_{{\rm{20min}}}}) + 38.726] \times 5.5}}$$

## Discussion

The therapeutic effect of the ionising radiation of ^131^I applied in the course of hyperthyroidism treatment depends above all on the dose of radiation absorbed by the thyroid gland. This statement is one of the key points of the standards regarding radioiodine treatment for hyperthyroidism issued by the EANM. The EANM guidelines mention the ranges of absorbed doses of ionising radiation according to the type of hyperthyroidism. In patients with autonomous nodules, the recommended dose is 300–400 Gy. In patients with GD, the dose with the aim of restoring a euthyroid status is approximately 150 Gy, whereas the dose to achieve complete ablation is in the range 200–300 Gy. For calculating the therapeutic activity of radioiodine, the EANM guidelines recommend the Marinelli formula. Apart from the aforementioned absorbed dose, the formula requires the evaluation of maximum thyroidal iodine uptake and of the effective half-life of radioiodine in the thyroid [1]. In an accurately prepared GD patient, the effective half-life has—according to our calculations—a steady value of 5.5 days on average [7–10]. This is why we are convinced that Marinelli’s formula can be simplified by substituting *T*_eff_ with 5.5 days.

In their attempts to streamline the calculation of the therapeutic activity of radioactive iodine using Marinelli’s formula, some authors resort to a variety of methods to determine the value of maximum thyroid iodine uptake. As a rule, the maximum uptake occurs after 24 h of the administration of radioactive iodine (^131^I) [10–12]. Morris and Hayes et al. propose algorithms for predicting ^131^I uptake through measuring it after 4 or 6 h, which considerably shortens the time of the procedure. In Morris’ study, the difference between the actual 24 h measurement and the estimated value after 4 h amounted to +10 %, and +5.9 % after 6 h. Hayes et al., meanwhile, proved that the correlation coefficient between the actual measurements and the 4- and 6-h estimates was high, reaching *r* = 0.94 [13, 14].

Still another approach to the problem can be found in the research results published by authors such as Shapiro or Yaqub. In both studies the maximum thyroid uptake in GD patients was calculated—as it usually is—after 24 h, but it was done by means of ^123^I instead of ^131^I. ^123^I possesses better physical parameters for isotope diagnostics (^123^I, in contrast to ^131^I, is a pure emitter of gamma radiation, characterised by a lower energy level and shorter half-life) [15, 16]. Osaki et al. combined the above two approaches to calculating IU_max_ based on Marinelli’s formula by assuming that it represented by the value of 24 h thyroidal iodine uptake and can be predicted using the ^123^I uptake after 3 h. They claim in their publication that the algorithm they propose for determining the correlation between the 3- and the 24-h thyroidal iodine uptake is statistically significant (*p* < 0.001) and can be used in clinical practice [8]. It has to be noted, however, that as for practical application, this method has the distinct disadvantage of being expensive, especially in Poland. The costs of producing the cyclotron-based iodine isotope ^123^I far outweighs those of acquiring the widely used reactor-produced isotope of iodine, ^131^I.

Smith et al. present yet another strategy for assessing IU_max_ in the thyroid, although their research was conducted on patients with non-toxic goitre, not with GD as in the case of the previously mentioned authors. Departing from an analysis of the measurements of thyroid ^99m^Tc and ^131^I uptake in each of their 44 patients, Smith et al. created an algorithm estimating the 24-h thyroid radioactive iodine uptake based on the level of ^99m^Tc uptake at 5 min of administering the radiomarker. They also emphasise that assessing iodine uptake on the basis of technetium scintigraphy allows for the greatest reduction of the patients’ exposure to ionising radiation. The thyroid absorbed dose of radiation emitted by technetium is 10 times as low as it is when ^123^I is used, and 10,000 lower than in the case of treatment with ^131^I. Also the effective dose of ionising radiation received by the entire organism is markedly lower when technetium is used for testing instead of ^123^I or ^131^I iodine [17]. The exposition from the diagnostic activity of radioiodine is unimportant considering the dose received from the following radioiodine therapy. Our study uses similar assumption as regards the prediction of thyroidal iodine uptake on the basis of technetium uptake, the only difference being that our sample consisted of patients with GD. The choice of study sample was determined by the fact that in GD the thyroid tissue is usually homogeneous and there are no problems associated with the occurrence of nodules that take up ^99m^Tc and ^131^I in different ways, as it is sometimes the case in a nodular goitre. These discrepancies result from the lack of ^99m^Tc organification by thyrocytes, which occurs when ^131^I is used [18]. Had we additionally taken into consideration the measurements of uptake values for the nodular goitre, the study sample would have become overly heterogeneous, making the calculations imprecise.

According to our investigations, the algorithm that depicts the correlation between the uptakes of ^131^I and ^99m^Tc is determined by the level of TRAb antibodies. The level of TRAb that significantly changes the algorithm equals 10 IU/ml. In clinical practice, this means that when thyroidal iodine uptake is estimated in GD patients on the basis of ^99m^Tc uptake, the level of TRAb antibodies should be taken into account so as to apply the correct algorithm.

In conclusion, predicting the thyroidal uptake of ^131^IU_24h_ in GD on the basis of measuring ^99m^TcU_20min_ uptake is possible, thanks to the equation created in the course of our research. The application of this algorithm considerably shortens the time necessary for pre-therapy diagnostic procedures and makes it possible to calculate the therapeutic activity of radioactive iodine with the use of Marinelli’s formula, as recommended by the EANM.
